# Author Correction: Next-generation seismic model of the Australian crust from synchronous and asynchronous ambient noise imaging

**DOI:** 10.1038/s41467-023-38270-6

**Published:** 2023-05-09

**Authors:** Yunfeng Chen, Erdinc Saygin, Brian Kennett, Mehdi Tork Qashqai, Juerg Hauser, David Lumley, Mike Sandiford

**Affiliations:** 1 Key Laboratory of Geoscience Big Data and Deep Resource of Zhejiang Province, School of Earth Sciences, Zhejiang University, Hangzhou, 310027 China; 2 Deep Earth Imaging, Future Science Platform, CSIRO, Kensington, WA 6151 Australia; 3 Research School of Earth Sciences, Australian National University, Canberra, ACT 2601 Australia; 4 Departments of Geosciences, Physics, University of Texas at Dallas, Richardson, TX USA; 5 School of Earth Sciences, University of Melbourne, 3010 Melbourne, Australia

**Keywords:** Seismology, Tectonics

Correction to: *Nature Communications* 10.1038/s41467-023-36514-z, published online 02 March 2023

The original version of this Article contained an error in the captions of Figures 2, 3, and 4, in which the major tectonic boundaries incorrectly referred to reference 47. The correct version refers to reference 48 in place of 47. Reference 48 has been added as

48. Korsch, R. J. & Doublier, M. P. Major crustal boundaries of Australia, and their significance for mineral systems targeting. *Ore Geol. Rev.* **76**, 212–228 (2016).

Original reference 48 (Hughes, A. Australian Operating Mines Map 2019. http://pid.geoscience.gov.au/dataset/ga/133033 (Geosciences Australia, 2020)) has been updated to reference 49.

This has been corrected in both the PDF and HTML versions of the Article.

