# Supplementary material for: Next-generation seismic model of the Australian crust from synchronous and asynchronous ambient noise imaging
Source: Nat Commun. 2023 Mar 2;14:1192. doi: 10.1038/s41467-023-36514-z (PMC9981728; doi:10.1038/s41467-023-36514-z)
Supplement: Supplementary file 1 — Supplementary Information [file 41467_2023_36514_MOESM1_ESM.pdf]

Supplementary Information

Chen et al.

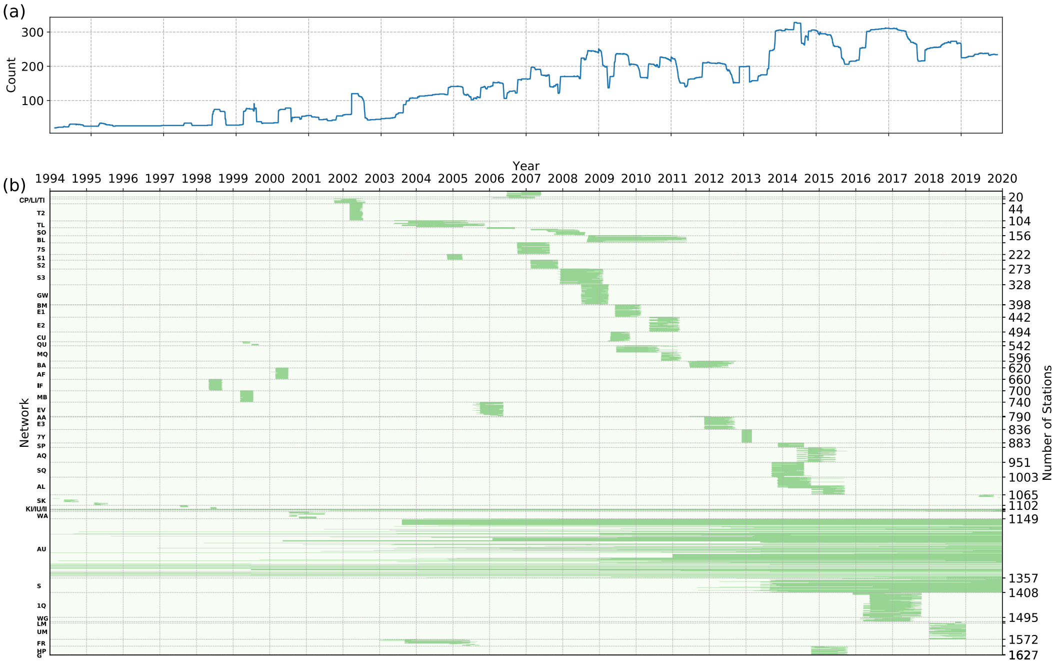

Supplementary figure 1. Temporal distribution of seismic stations used in this study. (a) The number of stations in each year. (b) The active period is shaded in green. The names of networks are labeled on the left of vertical axis. The numbers on the right side indicate the cumulative amount of stations in the joint network. Note that most of the stations are from portable arrays with limited operating time.

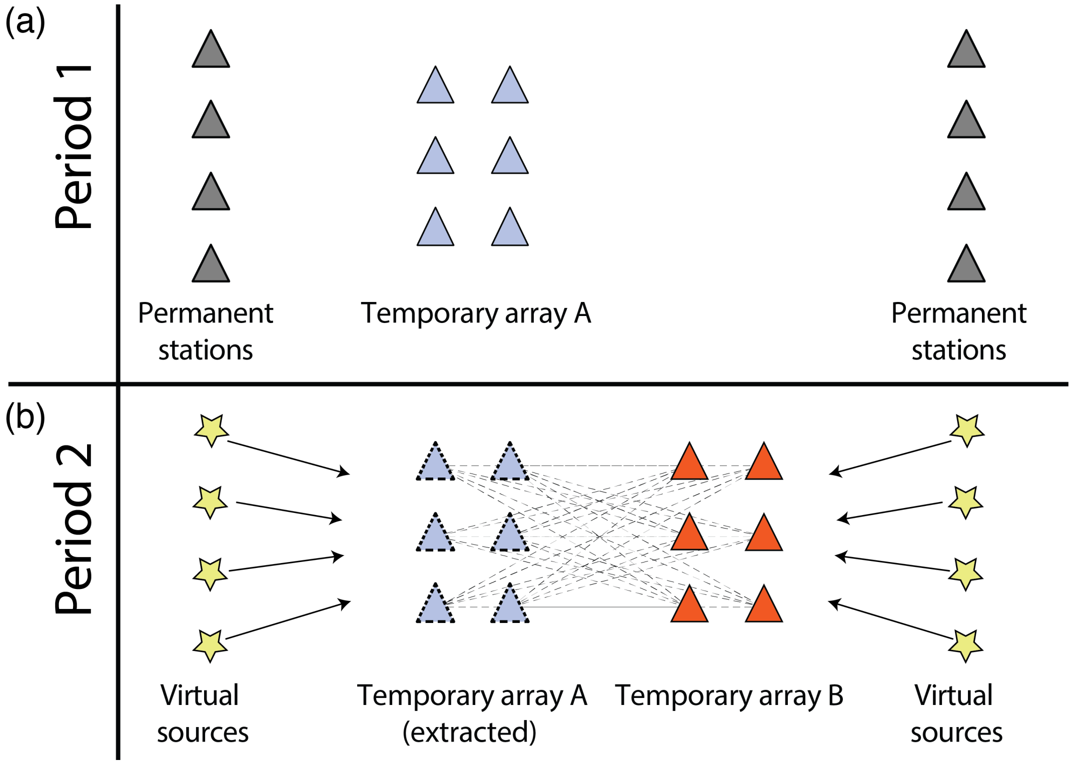

Supplementary figure 2. Cartoon illustrates the concept of C^2. Temporary arrays A and B are deployed at different periods and are surrounded by long operating stations from permanent networks. The C^2 method effectively turns the permanent stations into virtual sources that enable reconstructing the NCFs between stations from the two arrays.

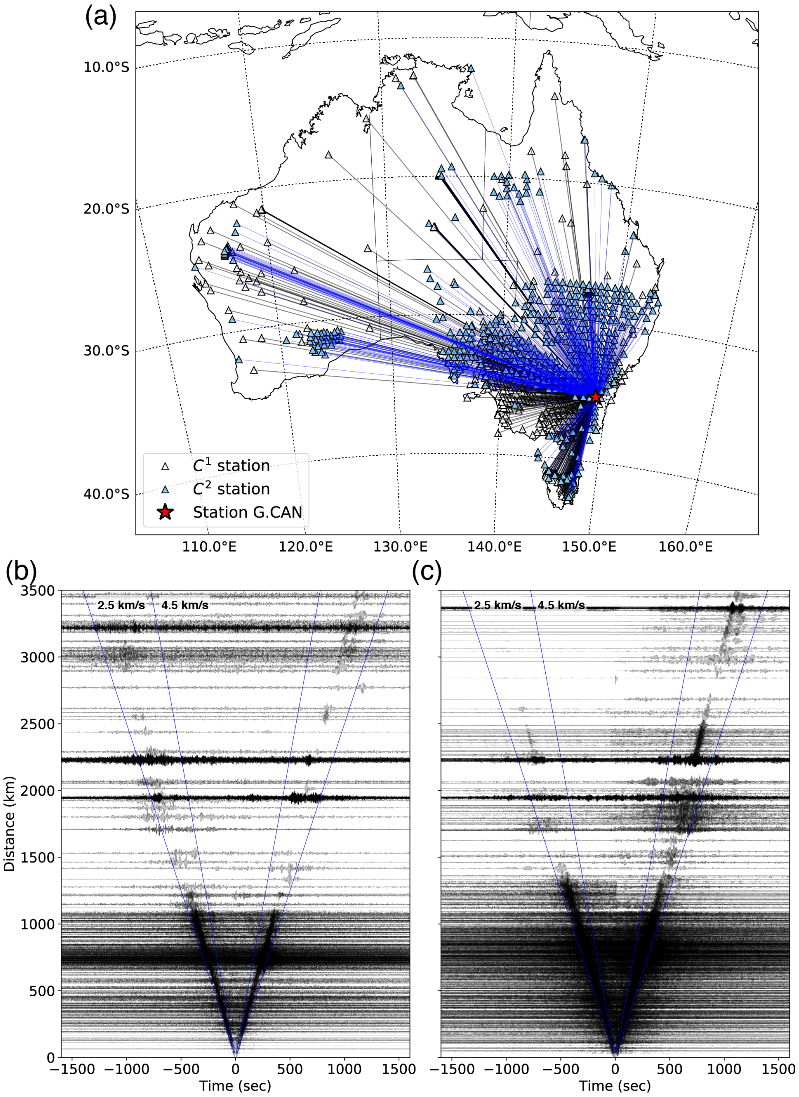

Supplementary figure 3. (a) Ray paths of C^1 and C^2 from station G.CAN in southeast Australia. Its deployment is concurrent with dense arrays in southeast Australia and long operating stations across the continent. Waveforms of (b) C^1 and (c) C^2 functions. The ray paths of C^1 and C^2 are shown in black and blue, respectively.

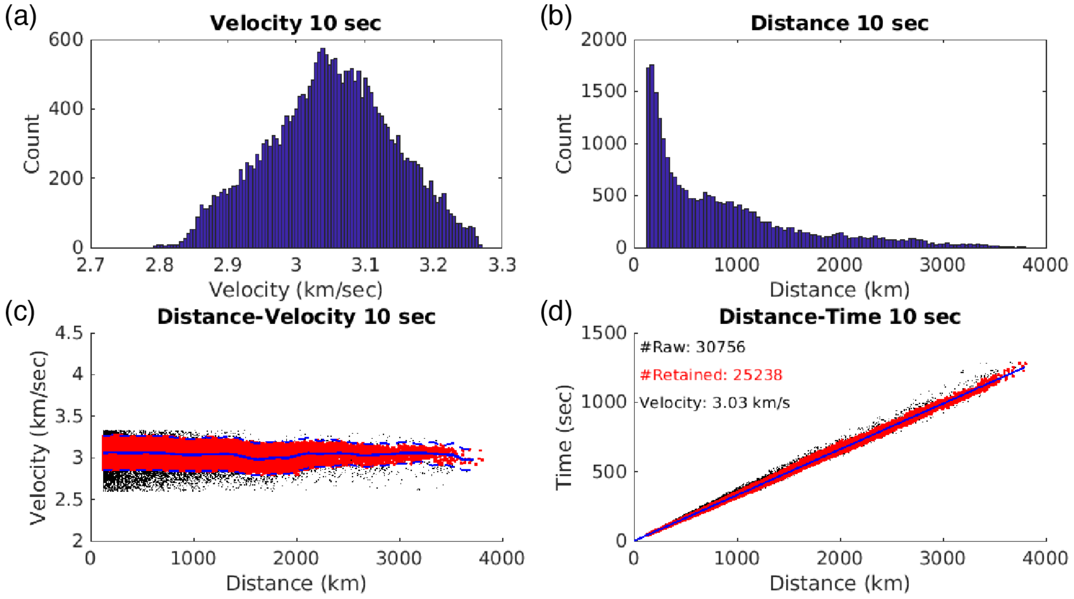

Supplementary figure 4. Data selection process. (a) Number of measurements as a function of velocity. (b) Number of measurements as a function of inter-station distance. The sharp cut-off at shorter distance is caused by imposing a minimum distance criteria. (c) Distribution of velocity as a function of distance. The black circles show all measurements. The solid blue line shows the running mean of the scattered points and the blue dashed line shows the standard deviation. The measurements within one standard deviation are retained (red circles). (d) The distance-time relationship. The black and red circles correspond to those shown in (c). The blue line represents the linear trend obtained by fitting all red points.

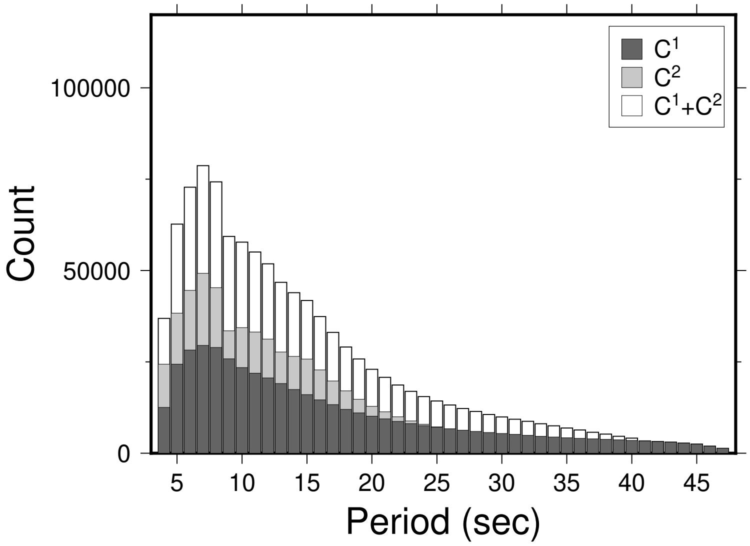

Supplementary figure 5. Number of robust group velocity dispersion measurements for C^1 and C^2 functions at all periods.

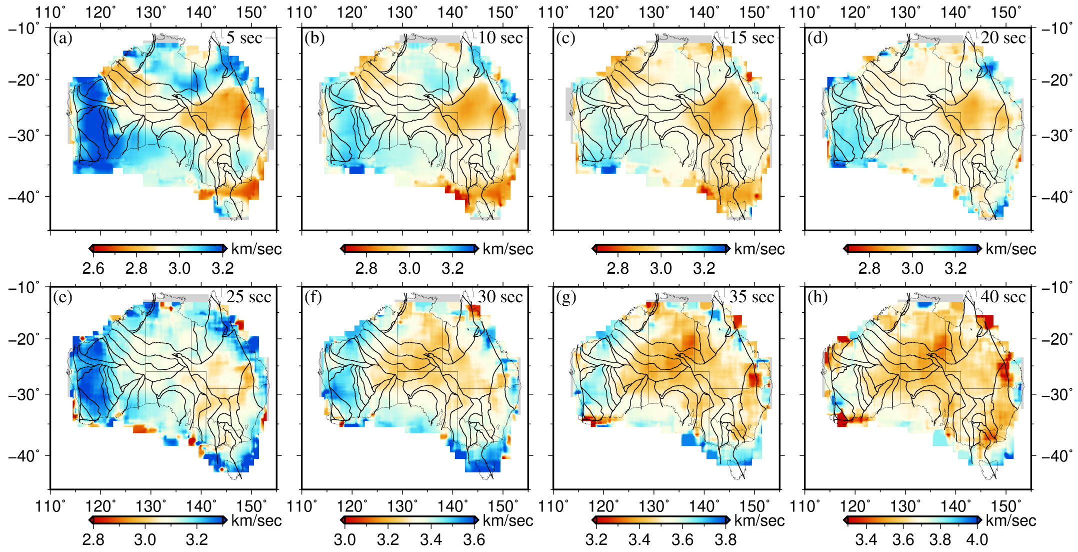

Supplementary figure 6. Cap-averaged group velocities at eight selected periods. The map is constructed by placing the average group velocity at the midpoint of each ray path and then smoothed with a 1.25-deg moving window in both directions.

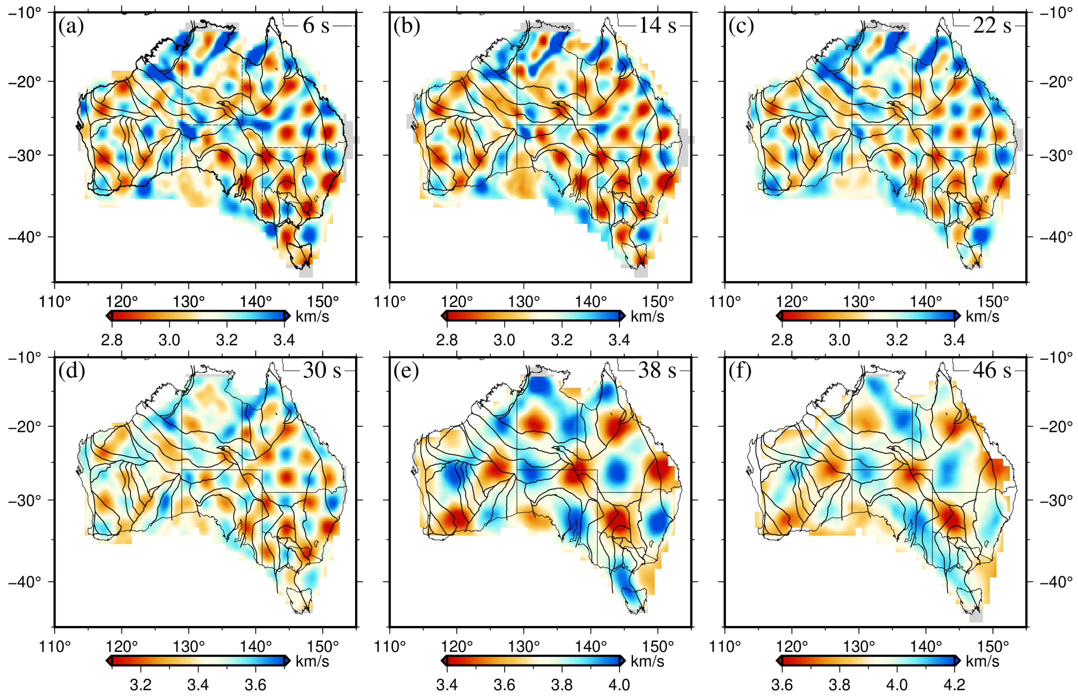

Supplementary figure 7. Checkerboard test results at six selected periods. The input structures consists of alternating velocity anomalies of about (a-d) 1.5 deg and (e-f) 3.0 deg.

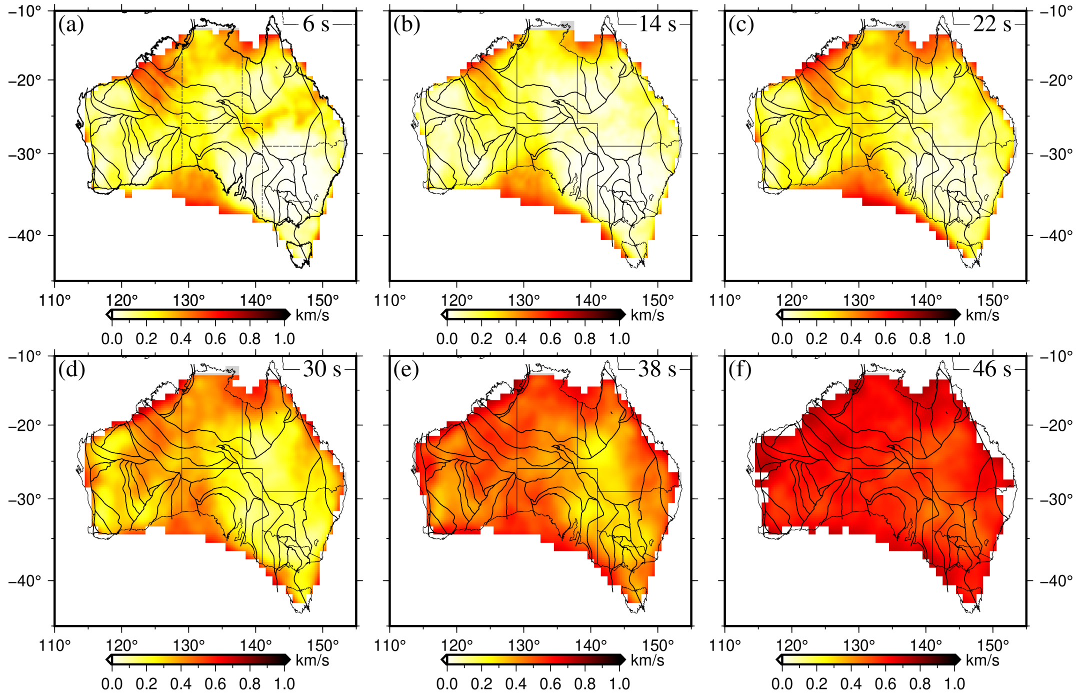

Supplementary figure 8. Model variances from trans-dimensional tomography at six selected periods.

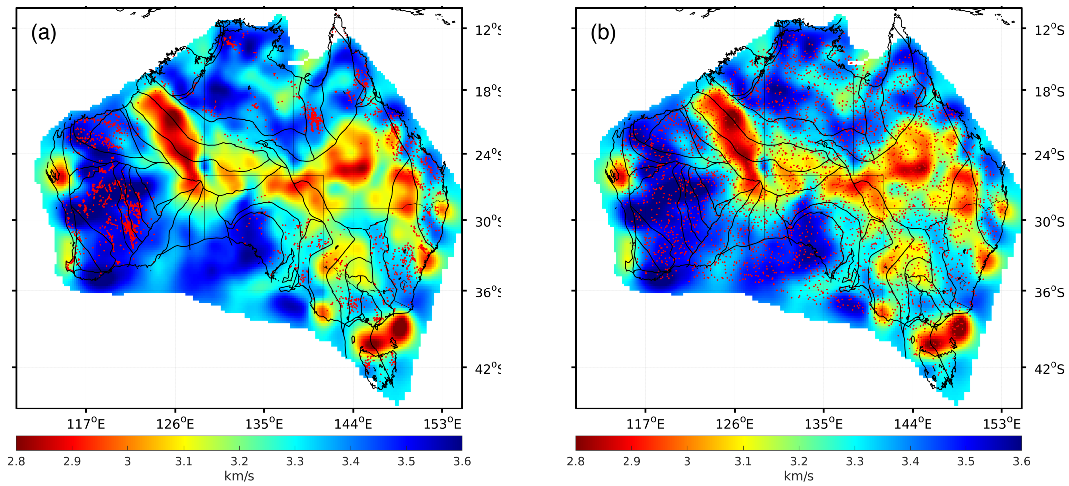

Supplementary figure 9. Spatial relationship between average velocities in the depth range of 0-5 km and 3975 mineral deposits (red circles) with (a) observed locations and (b) randomly assigned locations from one trial.

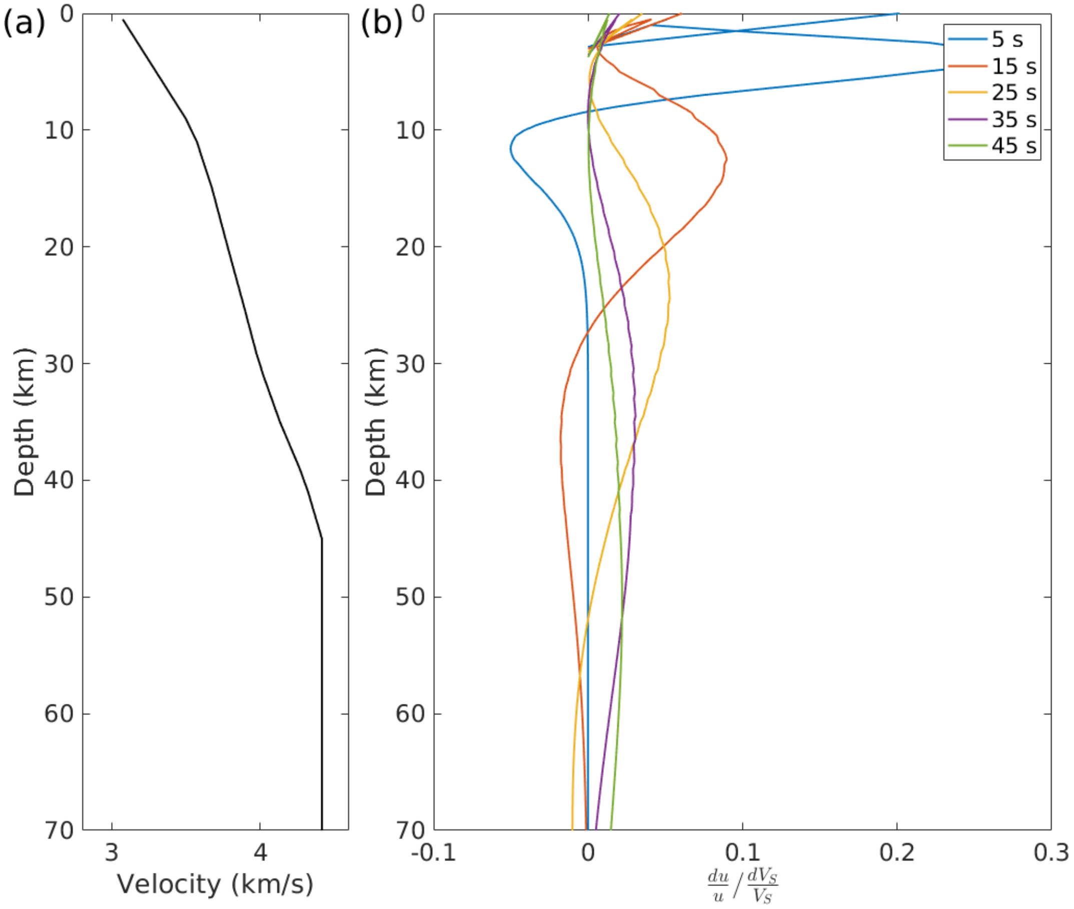

Supplementary figure 10. Velocity model obtained by averaging the AusREM model and is used to compute (b) group velocity sensitivity kernel.

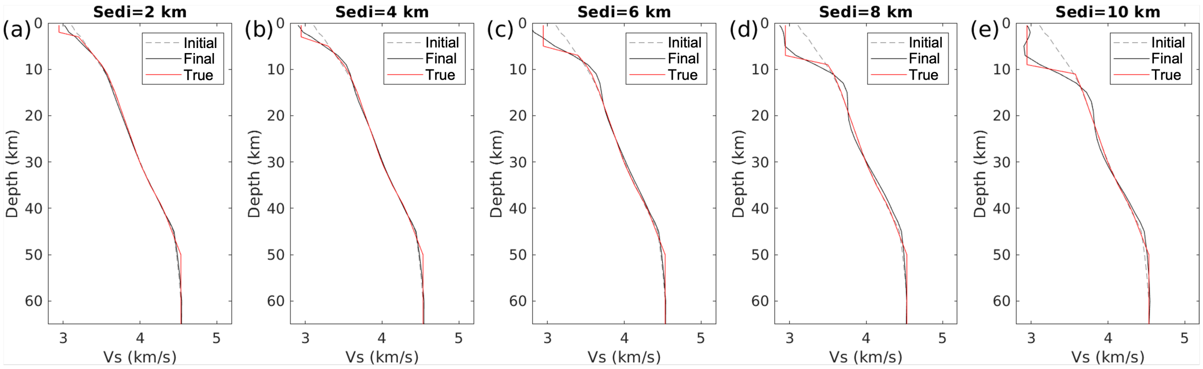

Supplementary figure 11. Inversion of 1D velocity structure with varying sediment thickness.

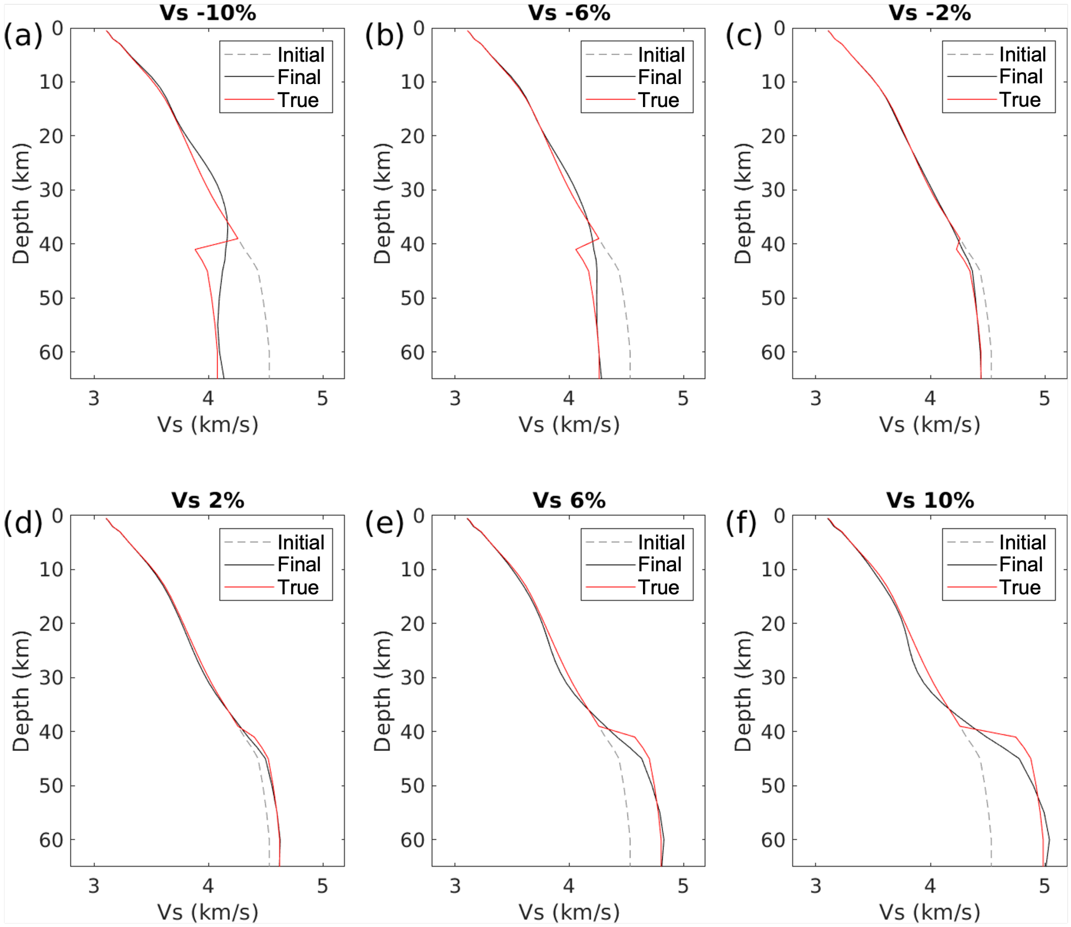

Supplementary figure 12. Inversion of 1D velocity structure with varying velocity perturbations in upper mantle.

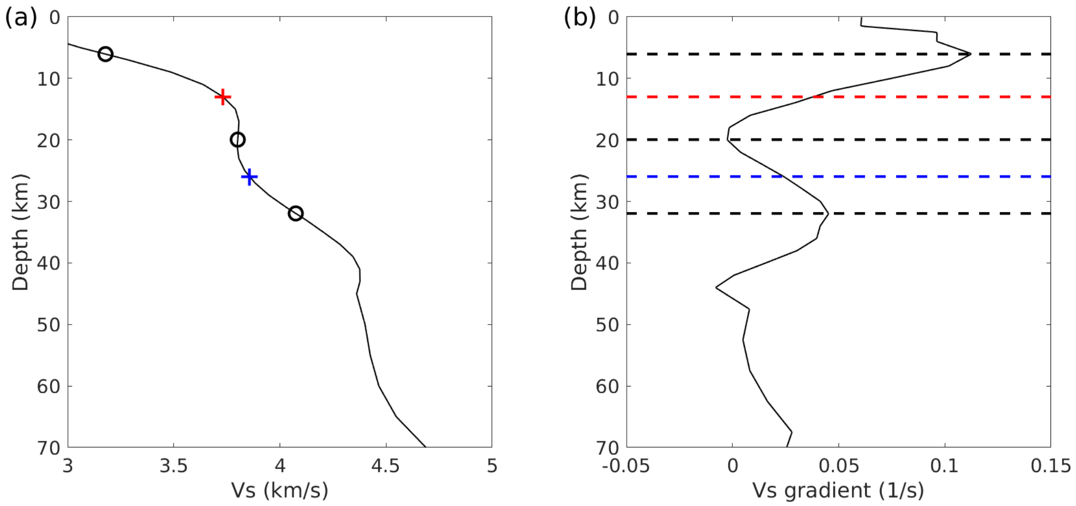

Supplementary figure 13. An example measurement of crustal interfaces. (a) A 1D shear velocity profile. The black circles indicate the depths where the velocity gradient reaches local extrema. The red and blue crosses indicate the depth of the upper-middle and middle-lower crustal boundaries, respectively. (b) The velocity gradient of shear velocity profile shown in (a). The depths of gradient extrema and crustal boundaries are indicated by the dashed lines in corresponding colors.

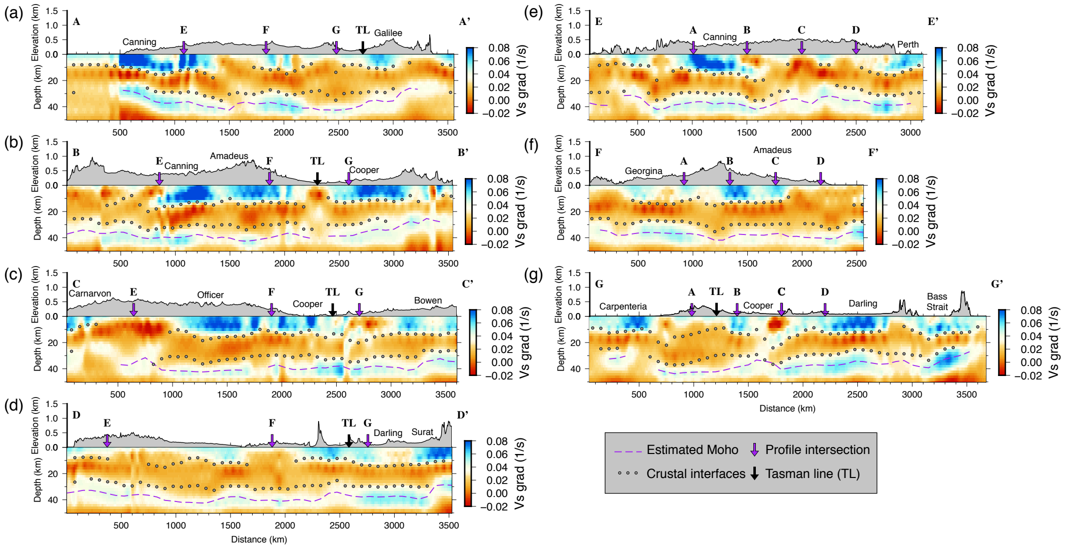

Supplementary figure 14. The cross-sections of our model showing vertical velocity gradients. The locations are shown in Figure R1. The gray circles represent major crustal interfaces. The Moho extracted from our model is indicated by the purple dashed line.

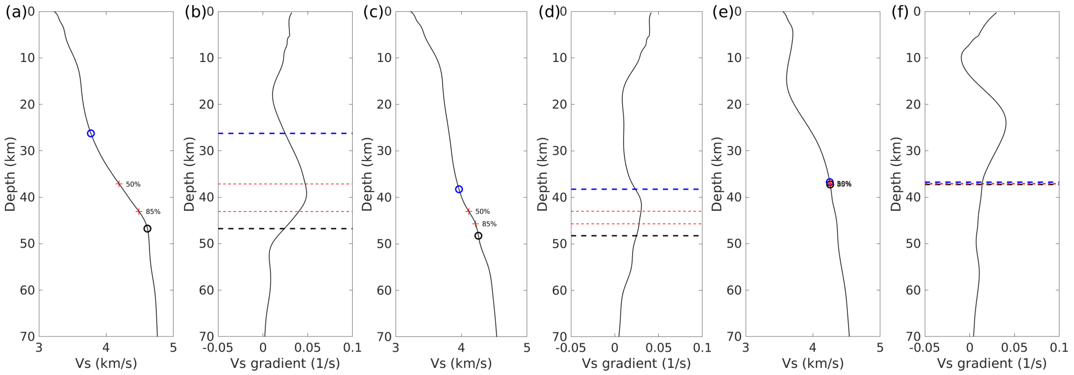

Supplementary figure 15. Representative velocity profiles with (a) thick, (c) thin and (e) undefined Moho transition thickness. The blue and black circles mark the depths of lower crust and upper mantle velocities. The red crosses mark 50% and 85% velocity jump from lower crust to upper mantle. (b, d, f) The corresponding velocity gradient profile of shear velocity. The dashed lines in corresponding colors mark the depths determined from shear velocities.

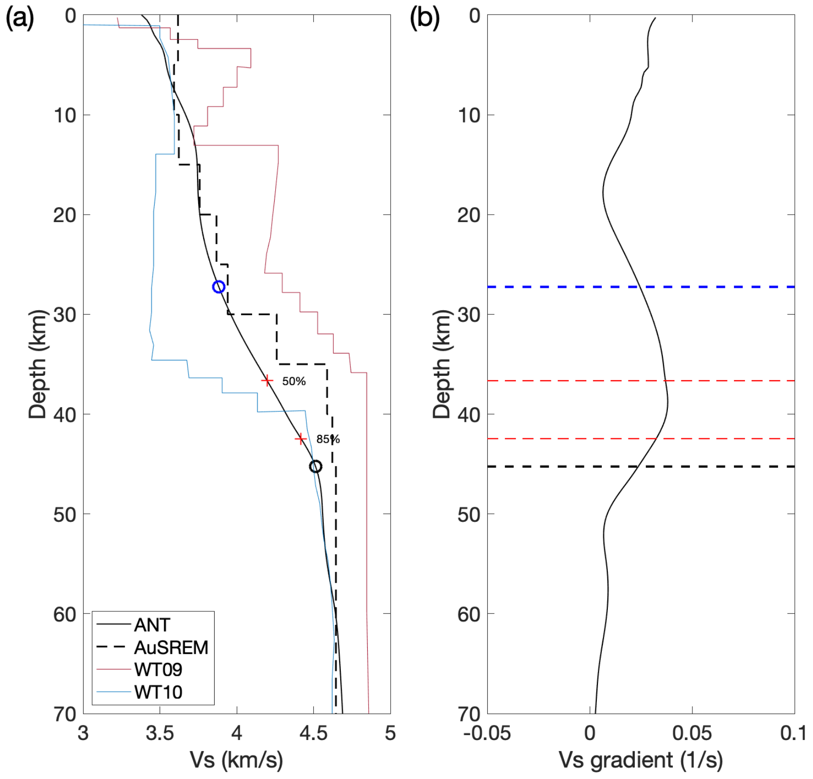

Supplementary figure 16. (a) Comparison of shear velocity profiles between our model and receiver function results from two stations WT09 and WT10 deployed in eastern Yilgarn craton[1]. (b) Shear velocity gradient from our model.

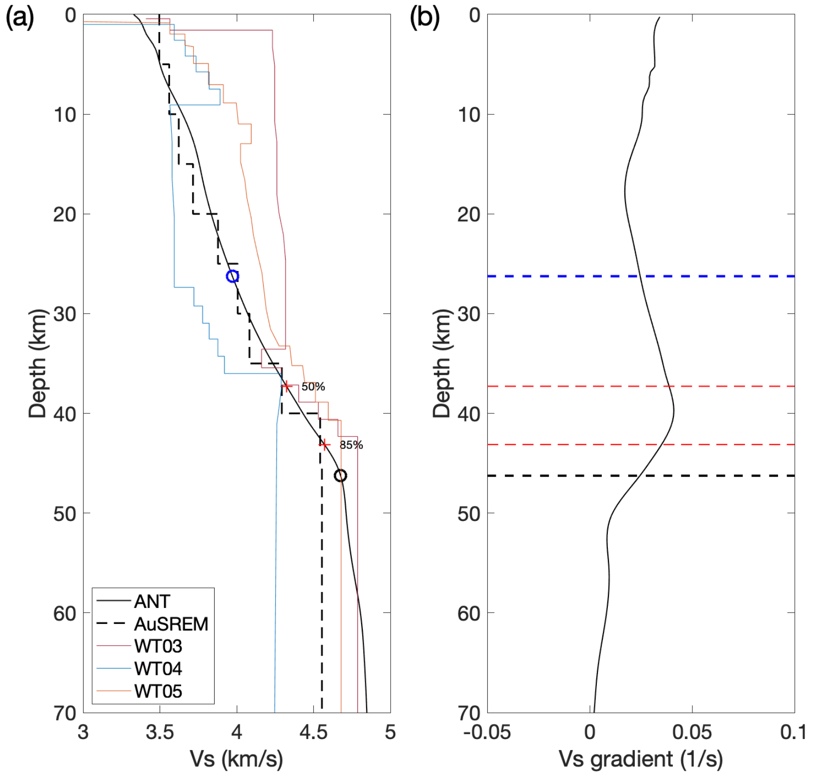

Supplementary figure 17. Similar to Figure S16 but for three stations WT03, WT04, WT05 deployed in western Yilgarn craton[1].

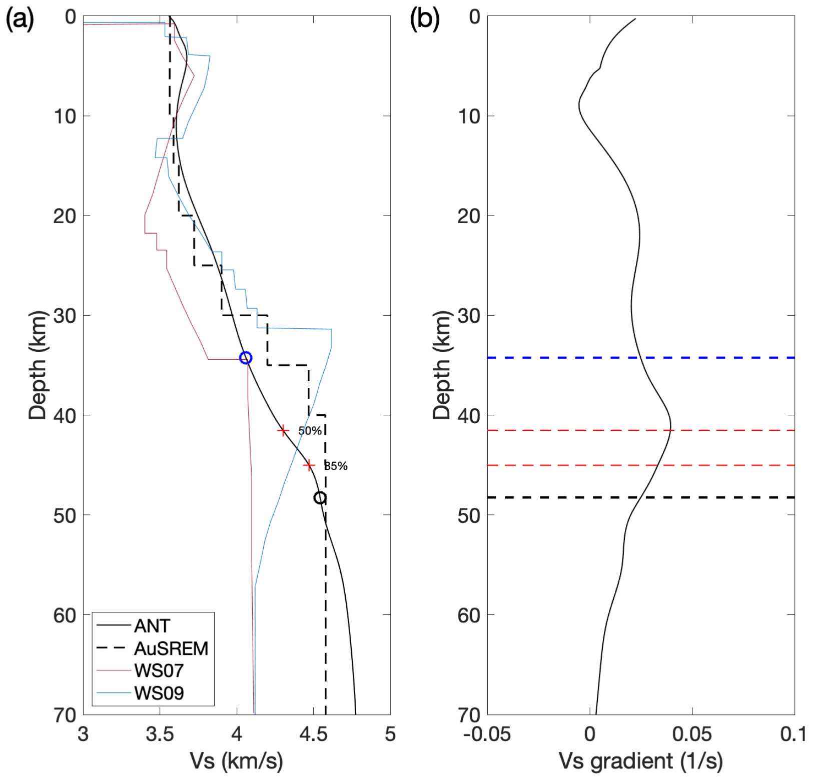

Supplementary figure 18. Similar to Figure S16 but for two stations WS07 and WS09 deployed in Pilbara craton[2].

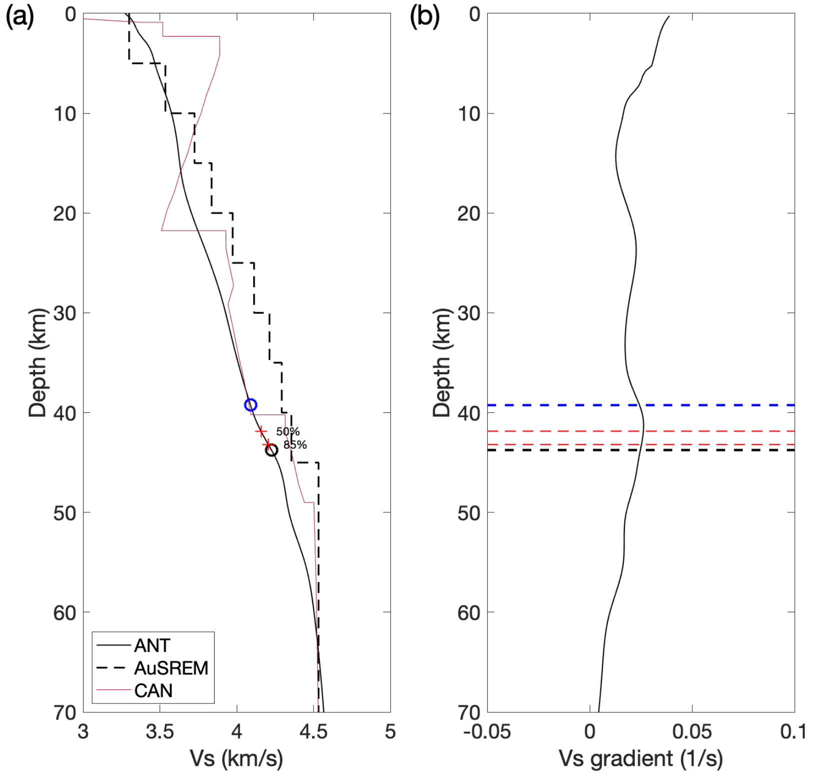

Supplementary figure 19. (a) Similar to Figure S16 but for station CAN deployed in southeastern Australia[3].

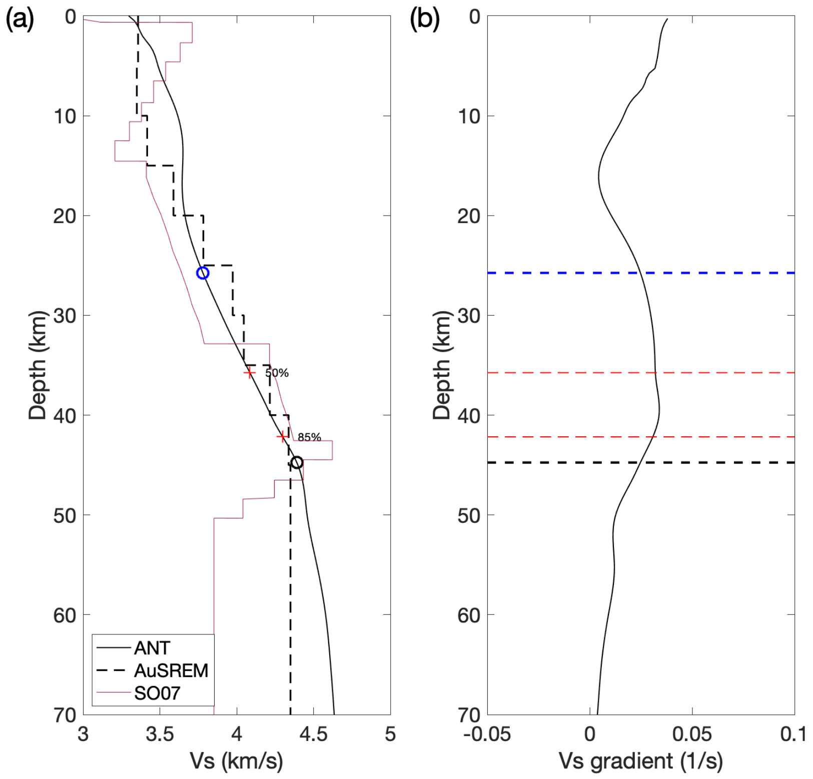

Supplementary figure 20. Similar to Figure S16 but for station SO07 deployed in southeastern Australia[4].

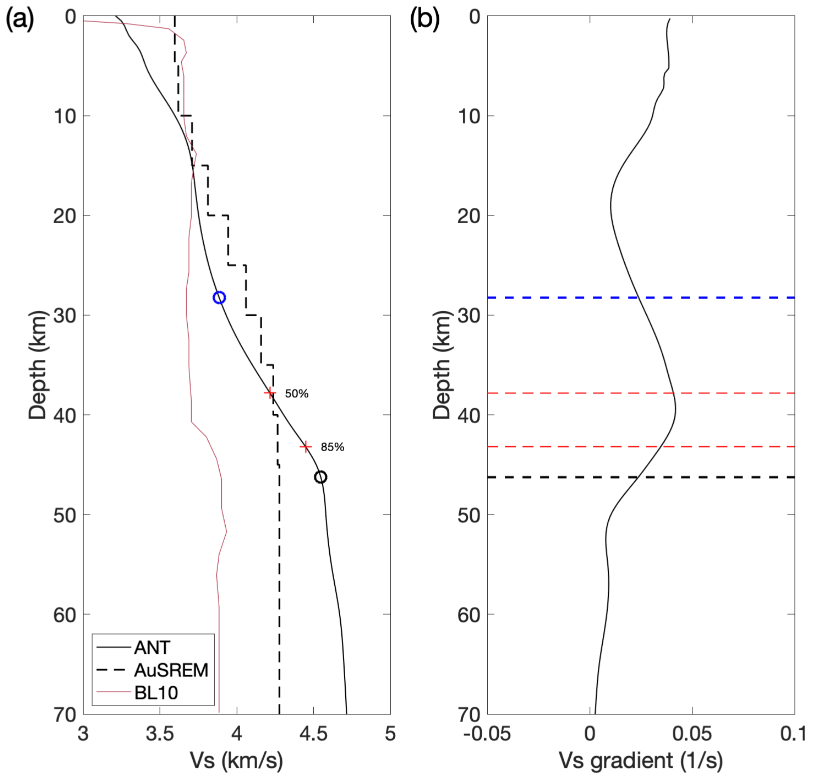

Supplementary figure 21. (a) Similar to Figure S16 but for station BL10 deployed in central Australia[5].

Supplementary References

1. Reading, A., Kennett, B. & Dentith, M. Seismic structure of the Yilgarn craton, Western Australia. *Australian Journal of Earth Sciences* **50**, 427–438 (2003).

2. Reading, A. & Kennett, B. Lithospheric structure of the Pilbara Craton, Capricorn Orogen and northern Yilgarn Craton, Western Australia, from teleseismic receiver functions. *Australian Journal of Earth Sciences* **50**, 439–445 (2003).

3. Bello, M., Cornwell, D. G., Rawlinson, N., Reading, A. M. & Likkason, O. K. Crustal structure of southeast Australia from teleseismic receiver functions. *Solid earth* **12**, 463–481 (2021).

4. Fontaine, F. R., Tkalčić, H. & Kennett, B. L. Imaging crustal structure variation across southeastern Australia. *Tectonophysics* **582**, 112–125 (2013).

5. Sippl, C. Moho geometry along a north–south passive seismic transect through Central Australia. *Tectonophysics* **676**, 56–69 (2016).
